# Initial experience and favorable outcomes on cannulation strategies and surgical platform construction in fully video-assisted thoracoscopic cardiac surgery

**DOI:** 10.3389/fcvm.2024.1414333

**Published:** 2024-08-08

**Authors:** Zihou Liu, Abulizi Maimaitiaili, Xiaozhong Ma, Shuangfeng Dong, Wei Wei, Qiang Wang, Qingliang Chen, Jianshi Liu, Zhigang Guo

**Affiliations:** ^1^ Department of Cardiovascular Surgery, Clinical School of Thoracic, Tianjin Medical University, Tianjin, China; ^2^ Department of Cardiovascular Surgery, Chest Hospital, Tianjin University, Tianjin, China; ^3^ Department of Cardiothoracic Surgery, Renmin Hospital of Hotan Prefecture, Xinjiang Uygur Autonomous Region, Xinjiang, China

**Keywords:** minimally invasive cardiac surgery, fully video-assisted thoracoscopy, cannulation strategies, valve surgery, congenital heart disease repair

## Abstract

**Background:**

Minimally invasive cardiac surgery offers numerous advantages that patients and surgeons desire. This surgical platform encompasses cannulation strategies for cardiopulmonary bypass, optimal surgical access points, and high-quality visualization techniques. Traditional peripheral cannulation methods, though convenient, possess inherent limitations and carry the potential for complications such as retrograde dissection, stroke, or neurologic sequelae. Conversely, central cannulation may be ideally suited to circumvent the disadvantages above. Fully video-assisted thoracoscopy cardiac surgery represents a state-of-the-art platform, offering surgeons an unparalleled surgical view. This analysis aimed to delineate the efficacy and safety of transthoracic central cannulation strategies and the surgical platform during fully video-assisted thoracoscopy cardiac surgery.

**Methods:**

Between October 2022 and February 2024, we identified a cohort of 85 consecutive patients with cardiopulmonary bypass undergoing fully video-assisted thoracoscopy cardiac surgery at our institutions. The patients' mean age was 41.09 ± 14.01 years, ranging from 18 to 75 years. The mean weight was 64.34 ± 10.59 kg (ranging from 49 to 103 kg). Congenital heart disease repair accounted for the highest proportion, with 43 cases (50.59%). Mitral valve surgery and left atrium Myxoma resections accounted for 29.41%. Specifically, this included 14 mitral valve repairs, five mitral valve replacements, and six left atrium myxoma resections. Aortic valve replacements constitute 20% of all cases.

**Results:**

A total of 85 adult patients underwent fully video-assisted thoracoscopy cardiac surgery. The average CPB time was 83.26 ± 28.26 min, while the aortic cross-clamp time averaged 51.87 ± 23.91 min. The total operation time (skin to skin) averaged 173.8 ± 37.08 min. The mean duration of mechanical ventilation was 5.58 ± 3.43 h, ICU stay was 20.04 ± 2.83 h (ranging from 15.5 to 34 h), and postoperative hospital stay was 5.55 ± 0.87 days. No patients required conversion to thoracotomy and unplanned reoperations due to various reasons. There were no in-hospital deaths, strokes, myocardial infarctions, aortic dissections, or renal failure. No patient developed wound soft tissue infection.

**Conclusions:**

Fully video-assisted thoracoscopy cardiac surgery utilizing central cannulation strategies is a reliable, cost-effective platform with a low risk of complications and a potential solution for patients facing contraindications for peripheral cannulation.

## Introduction

Over the past few years, there has been a rapid advancement in minimal-invasive cardiac surgery (MICS). The advantages of this approach include superior cosmetic outcomes, reduced postoperative pain, blood loss, ICU and hospital stay, potential cost savings, and a shorter recovery time (1–5). Both surgeons and patients have expressed a strong interest in this procedure.

In the majority of experienced heart centers worldwide, cardiopulmonary bypass (CPB) with the femoral artery (FA) and vein cannulation, along with the percutaneous cannulation of the superior vena cava (SVC) via the internal jugular veins (IJV), has become the standard treatment for patients undergoing MICS (6, 7). However, there is an increasing risk and incidence of complications associated with femoral cannulation, including retrograde dissection, embolization, stroke, femoral arterial injury, and ipsilateral limb ischemia (5, 8–11). The percutaneous insertion of IJV cannulation typically requires an experienced anesthesiologist, which can be challenging to obtain in many general heart centers (7). Furthermore, unexpected vascular injuries can occasionally occur during the IJV cannulation, which may lead to severe consequences (12, 13). The exposure quality in MICS inversely correlates with procedure duration and the possibility of transitioning to a full sternotomy. Fully video-assisted thoracoscopic cardiac surgery (FVATCS) provides surgeons with a clear and magnified view of the heart and its surrounding structures (14, 15), and it represents a cutting-edge platform within the realm of MICS techniques.

This study aims to introduce and focus on meticulously designed cannulation strategies alongside a reliable FVATCS platform construction, avoiding repeating addressing the specific topics encompassed within FVATCS, including patient selection and preparation, technical details, considerations, and challenges related to mitral and aortic valve surgeries or diverse intra-cardiac procedures. This surgical platform features a fixed number of incisions and clearly defined and relatively stable incision positions.

## Methods

Between October 2022 and January 2024, we conducted a study on 85 consecutive patients who underwent MICS with CPB at our institutions. MIDCAB procedures were excluded from the study due to their off-pump execution. Following institutional review board approval, the study adhered strictly to the ethical guidelines outlined in the Declaration of Helsinki. Utilizing our institutional medical records, we gathered comprehensive data regarding patient demographics, surgical variables, and postoperative complications.

Preoperative computed tomography and ultrasound were routinely performed to detect aortic pathology and peripheral vascular disease. Anaesthesia was provided according to the standard protocol, and double-lumen endotracheal intubation was used to decompress the right lung. Transesophageal echocardiography was performed in all cases to assess the place of the venous cannulations and the surgical result. All patients were supine, with the right side of the body elevated 20°–30° and the right arm suspended above the head.

### Approach for congenital heart disease, tricuspid valve surgery, mitral valve surgery, and left atrium myxoma resections

**Figure 1:**
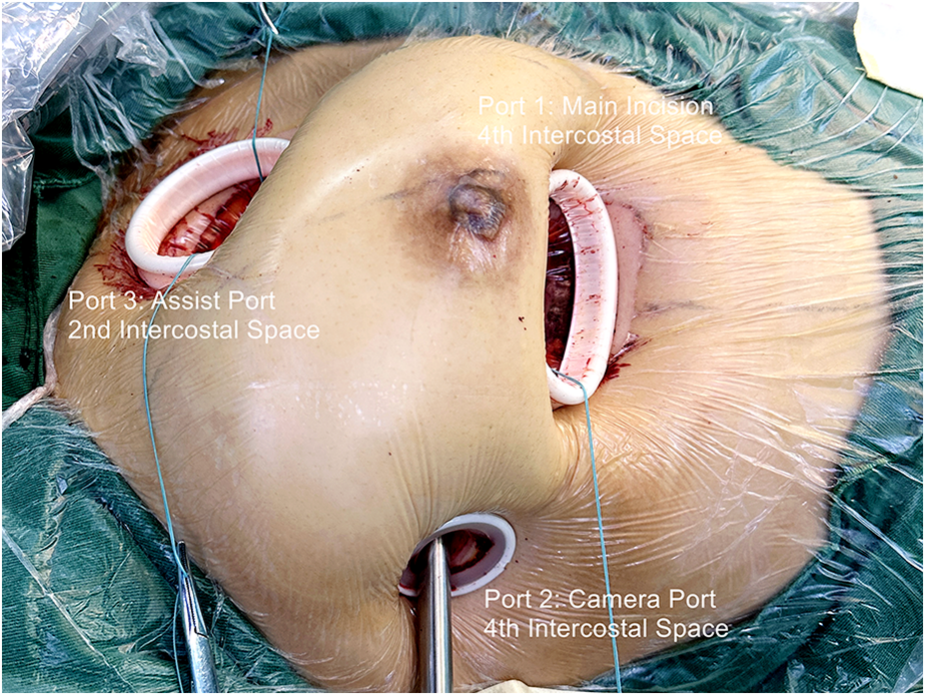
Fully video-assisted thoracoscopic cardiac surgery platform for congenital heart disease surgery, trispid valve surgery, or mitral valve surgery.

For congenital heart disease and tricuspid valve surgery, a 40 mm skin incision is made at the fourth intercostal space between the midclavicular and anterior axillary lines. In Mitral valve surgery and left atrium Myxoma resections, the incision is made between the anterior and midaxillary lines. This serves as the main operating port (port 1). For females, a semilunar incision is made at the lower edge of the right breast, fifth or sixth intercostal space, to avoid injury to the breast. The subcutaneous tissue is dissociated upward, and a thoracotomy is performed at the fourth intercostal space. The camera port (port 2) is positioned lateral to the main operating port in the same intercostal space, with a 10 mm skin incision. A 30 mm assist port (port 3) is inserted into the second intercostal space parasternal under thoracoscopic guidance while carefully avoiding damage to the right internal thoracic artery and intercostal arteries. Afterward, three small soft-tissue silicone retractors are inserted into the patient's body (Figure 1, Supplementary Video 1, Figure 1). A 10 mm oblique incision parallel to the inguinal ligament is made in the right groin to expose the femoral vein. This incision is typically positioned 15 mm above the inguinal crease.

Following systemic heparinization, a 24–28 Fr femoral vein cannula is inserted into the right atrium under the guidance of TEE utilizing the Seldinger technique. Thoracoscopic guidance makes a careful longitudinal incision in the pericardium, approximately 20 mm superior to the phrenic nerve. Subsequently, three or four sutures are placed to suspend the pericardium, fully exposing the ascending aorta and SVC (Supplementary Video 2).

The double concentric pursestring sutures are placed as high as possible on the anterolateral aspect of the ascending aorta via the main port and assist port incisions. The concentric pursestring suture on the SVC is routinely secured in place. Under induced hypotension (systemic arterial pressure <90 mmHg), the aorta is retracted down and right using a long vascular clamp, and an 18–22 Fr StraightShot aortic cannula is gently inserted directly toward the aortic arch. Subsequently, the cannula is carefully secured and put in place. An appropriately sized right-angled cannulation cannula for the SVC is inserted using a ringed clamp for assistance (Supplementary Video 3).

The aorta and SVC cannulation procedures are executed through port 3. Caval snares are positioned in the superior and inferior vena cava and exit the body through ports 3 and 2, respectively, for use in congenital heart disease and tricuspid valve surgeries.

**Figure 2:**
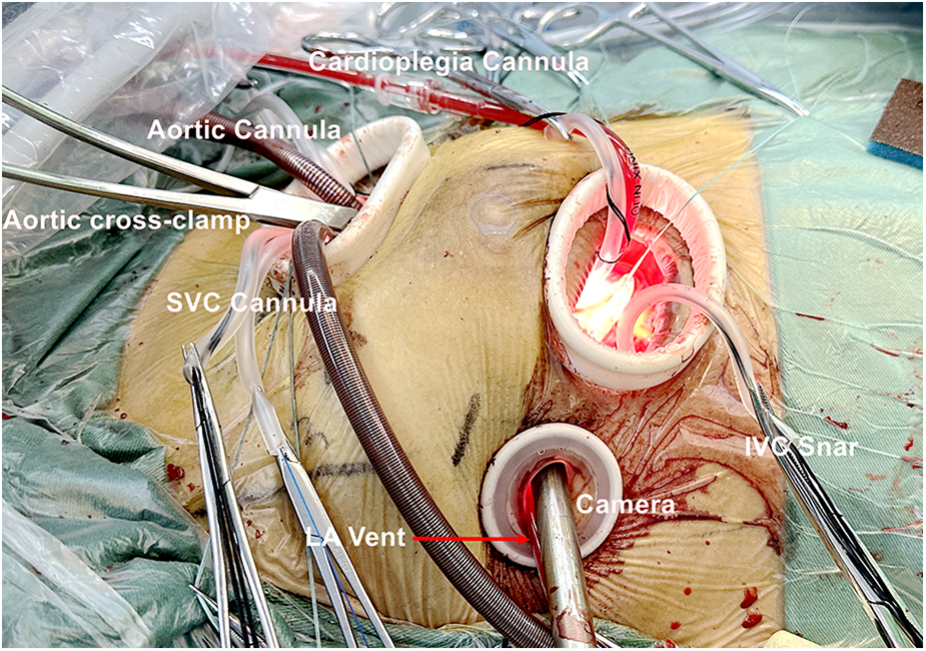
Cannulation in congenital heart disease surgery, trispid valve surgery or mitral valve surgery.

During the procedure, in order to achieve precision and safety, video and direct vision are used alternately, and special attention should be paid to avoid damage to the posterior wall of the aorta. The aortic perfusion needle is inserted into the aorta via port 1. The aorta is conventionally cross-clamped directly with care through port 3, and Del Nido cardioplegia is used for myocardial protection in all patients (Figure 2). The right atrium is opened from a site parallel to the atrioventricular annulus for congenital heart diseases and tricuspid valve surgery, and the mitral valve surgery and left atrium Myxoma resections are achieved through the interatrial groove. The left atrial vent cannula is inserted through port 2 and directly into the right superior pulmonary vein, and CO2 insufflation is initiated through port 3.

**Figure 3:**
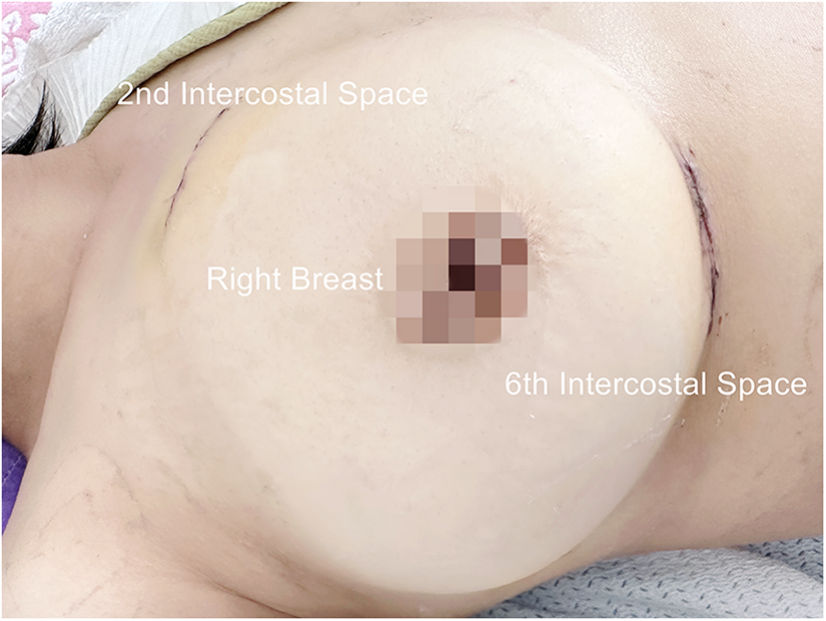
Surgical access after atrial septal defect and trispid valve repair.

Postoperatively, a pericardial drainage tube is inserted in the camera port (Figure 3).

### Approach for aortic root surgery

**Figure 4:**
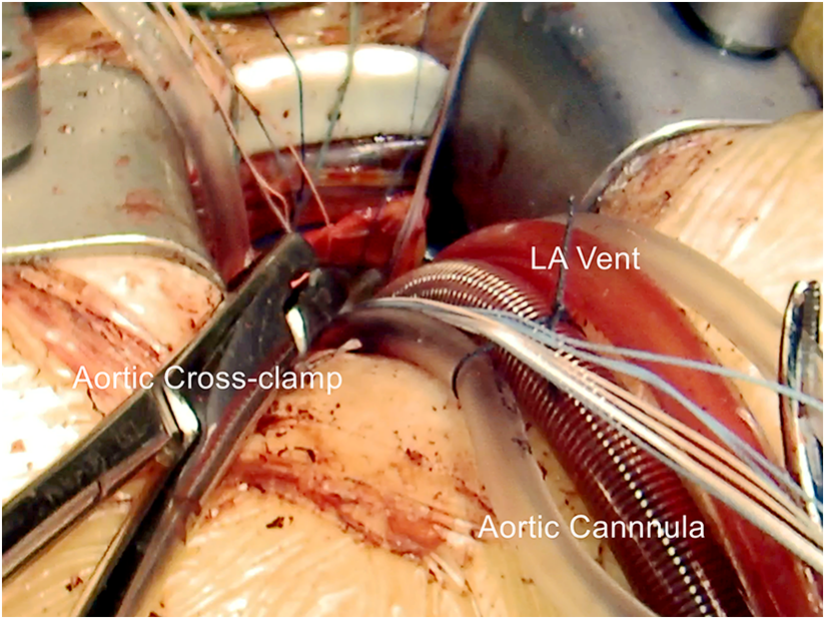
Cannulation in aortic valve surgery.

Aortic root surgery is performed via a 50–60 mm transverse incision (main incision) in the second right intercostal space. Following single lung ventilation, entry is made into the right pleural space. The right mammary artery and vein are left intact, and no ribs or costal cartilage are excised. Subsequently, a soft tissue retractor is inserted, followed by an intercostal rib spreader. An additional 10 mm incision is made for the assist port (camera port) in the second or third intercostal space, located laterally to the main port. Following heparinization, femoral vein cannulation was performed, followed by the insertion of a direct aortic cannula. The left ventricular vent is done through the main port and directed into the right superior pulmonary vein (Figure 4). The standard practice involves using aortic cross-clamp and Del Nido cardioplegia for all patients. When the aortic root needs access, a cannula directly administers the cardioplegic solution into each coronary ostium.

Once the patient's vital signs, including blood pressure and heart rate, stabilize and fulfill the discontinuation criteria for extracorporeal circulation, the upper vena cava cannula is initially withdrawn under thoracoscopic guidance, subsequently followed by the femoral vein cannula. Subsequently, blood is retransfused via the ascending aortic cannula. A small gauze pad is positioned at the insertion site of the ascending aortic cannula to mitigate blood spatter. Following cannula removal, the assistant promptly tightens the purse-string suture to contain bleeding and secures it with a double knot.

## Results

The primary outcome measures were short-term and long-term mortality. Short-term mortality was defined as death occurring within 30 days following surgery. Other outcomes of interest encompassed neurovascular complications and significant postoperative complications. Significant complications encompassed prolonged mechanical ventilation (>72 h), acute kidney injury, reoperation due to bleeding, and unplanned cardiac reoperation. The necessity for conversion to sternotomy was also documented as a safety endpoint.

**Figure 5:**
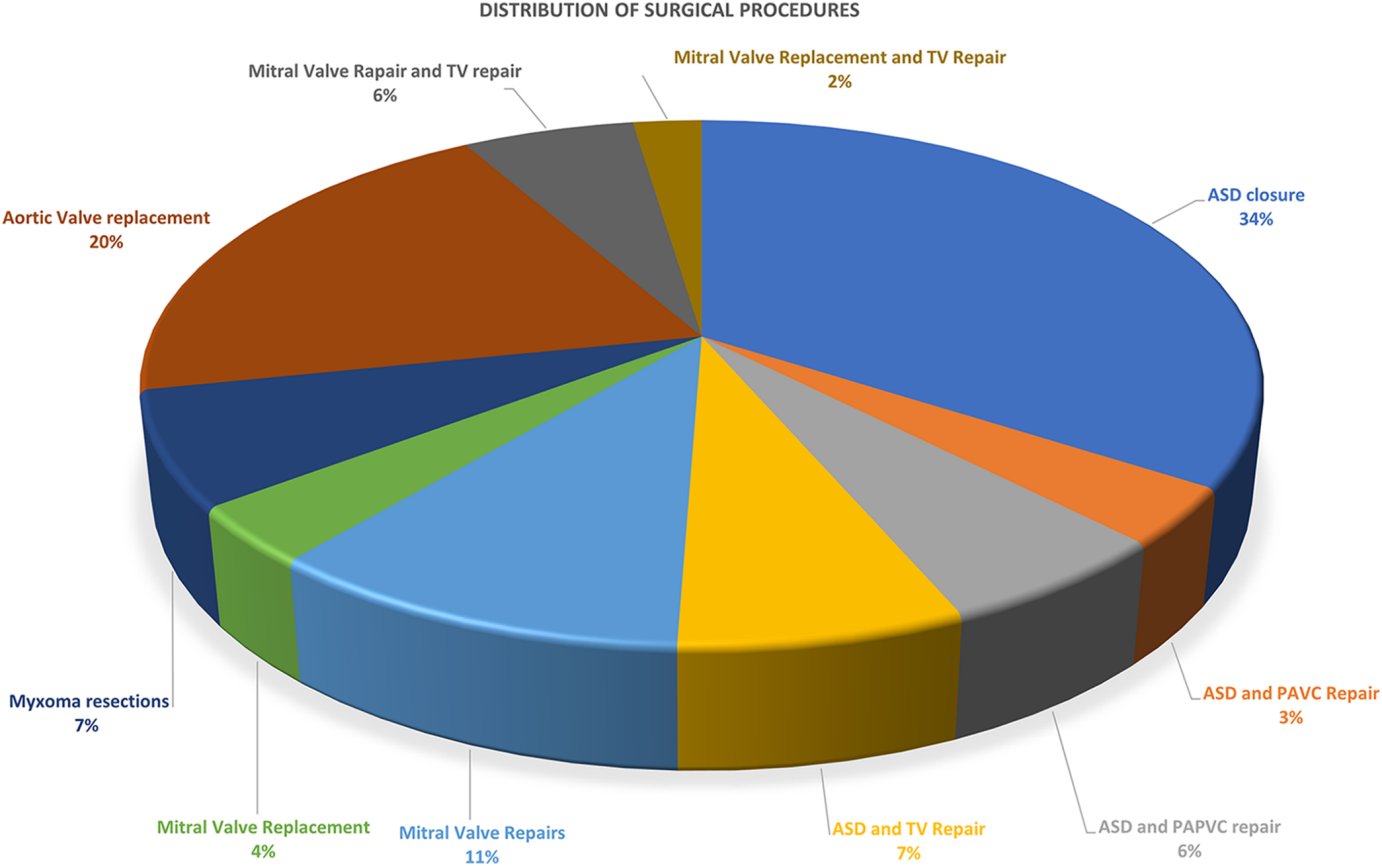
Pie chart showing the distribution of surgical procedures in total case.

Over the period from October 2022 to January 2024, we identified a cohort of 85 consecutive patients undergoing MICS with CPB at our institutions. Demographic details for the patients are presented in Table 1. The patients' mean age was 41.09 ± 14.01 years, ranging from 18 to 75 years. The mean weight was 64.34 ± 10.59 kg (ranging from 49 to 103 kg), while the mean BMI was 24.57 ± 3.03 (ranging from 20 to 39). Congenital heart disease repair accounted for the highest proportion of MICS operations, with 43 cases (50.59%). Mitral valve surgery and left atrium Myxoma resections were the subsequent most common surgeries, accounting for 29.41%. Specifically, this included 14 mitral valve repairs, five mitral valve replacements, and six left atrium Myxoma resections. The remaining surgeries were aortic valve replacements, constituting 20% of the total cases (Table 2, Figure 5 and Supplementary Figures 2–6).

**Table 1:** Preoperative demographics.

Variable	Value (mean ± sd)
Age, year	41.09 ± 14.01 (18–75)
Gender, female (%)	50 (58.82%)
Weight	64.34 ± 10.59 (49–103)
BMI	24.57 ± 3.03 (20–39)

BMI, body mass index.

**Table 2:** Procedural characteristics and operative results.

Variable	Value (mean ± sd or *n*)
CPB time, min	83.26 ± 28.26 (range 46–153)
Aortic cross-clamp time, min	51.87 + 23.91 (range 20–101)
Operation time (skin to skin), min	173.8 ± 37.08 (range 125–256)
Mechanical ventilation, hours	5.58 ± 3.43 (range 2–18.5)
ICU stay, hours	20.04 ± 2.83 (range 15.5–34)
Postoperative hospital stay, days	5.55 ± 0.87 (range 4–8)
Drainage, ml	285.14 ± 151.64 (range 100–710)
Associated procedures	
Congenital heart disease repair	**43**
ASD closure	29
PAVC repair	3
ASD and PAPVC repair	5
ASD and tricuspid valve repair	6
Mitral valve surgery and left atrium myxoma resections	**25**
Mitral valve repairs	9
Mitral valve replacement	3
Mitral valve repair and tricuspid valve repair	5
Mitral valve replacement and tricuspid valve repair	2
Left atrium myxoma resections	6
Aortic valve surgery	**17**
Aortic valve replacement	17
Total	**85**

CPB, cardiopulmonary bypass; ICU, intensive care unit; ASD, atrial septal defect; PAPVC, partial anomalous pulmonary venous drainage; PAVC, partial atrioventricular septal defect.

Bold values represent the number of surgical cases by surgical approaches.

The average CPB time was 83.26 ± 28.26 min (ranging from 46 to 153 min), while the aortic cross-clamp time averaged 51.87 ± 23.91 min (ranging from 20 to 101 min). The total operation time (skin to skin) averaged 173.8 ± 37.08 min (ranging from 125 to 256 min). The mean duration of mechanical ventilation was 5.58 ± 3.43 h (ranging from 2 to 18.5 h), ICU stay was 20.04 ± 2.83 h (ranging from 15.5 to 34 h), and postoperative hospital stay was 5.55 ± 0.87 days (ranging from 4 to 8 days). The drainage volume within the first 24 h was 285.14 ± 151.00 ml (ranging from 100 to 710 ml) (Table 2).

No patients required conversion to thoracotomy and unplanned reoperations due to various reasons. There were no in-hospital deaths, strokes, myocardial infarctions, aortic dissections, and renal failure. All the patients were routinely followed up at 1 month, 3 months, 6 months, 12 months, and yearly at discharge. Only one patient developed shallow wound soft tissue infection.

## Discussion

Conventional cardiac surgery typically employs a full sternotomy, enabling easy cannulation and perfusion strategies, optimal surgical visualization, and excellent outcomes (1). Nevertheless, the median sternotomy carries the risk of increased bleeding, postoperative discomfort, prolonged recovery time, residual hardware such as steel wires or plates, and a visible surgical scar. Minimally invasive cardiac surgery was performed through a lateral thoracotomy ranging from 4 to 7 cm in length, entirely eliminating the need for sternotomy, thereby promoting faster physical recovery, enhancing cosmetic outcomes, and minimizing postoperative complications and healthcare costs (3). Furthermore, numerous studies have established that MICS offers surgical quality comparable to traditional sternotomy procedures. The Society of Thoracic Surgeons (STS) database analysis and multicenter, propensity-score-matched study have demonstrated the safety equivalence between the minimally invasive approach and conventional methods, exhibiting low mortality and morbidity and superior immediate and long-term postoperative outcomes (16, 17).

In our series, the mean aortic cross-clamp time was 51 min, the CPB time averaged 83 min, and the mean overall operation time was 173 min. These durations highlight the feasibility and practicality of our cannulation strategies and surgical platform despite the initial learning curve encountered by introducing novel techniques. Meanwhile, no patient required conversion to a full sternotomy. Our results indicate that with time and experience, these techniques can seamlessly integrate into our surgical practice, ensuring reasonable operative durations. Furthermore, our study observed a shortened ICU stay and hospital length of stay, along with reduced postoperative bleeding. Notably, no deaths or complications were reported during the hospital stay or the follow-up period. We speculate that the observed phenomenon is closely associated with the cannulation strategies and the minimally invasive surgical platform we employed.

Multiple minimally invasive surgical techniques for MICS, including upper and lower hemisternotomy, right parasternal approach, and right mini-thoracotomy, have been established (7, 18, 19). The right mini-thoracotomy has emerged as the preferred incision for MICS, serving as the standard approach across numerous centers. Rather than being a solitary approach, MICS embodies diverse techniques. These techniques range from alternative cannulation strategies and modified aortic occlusion to video assistance visualization technology (20, 21). Peripheral cannulation, a straightforward and frequently utilized method for CPB, is commonly used in MICS performed via a right mini-thoracotomy (22, 23). Aortic occlusion can be achieved using two main techniques: external trans-thoracic cross-clamp (ETTC) and endo-aortic balloon occlusion (EABO).

However, several studies have suggested an association between FA cannulation with retrograde perfusion and increasing mortality and stroke or neurologic complications (8, 9, 24, 25). Analysis of the STS database reveals the rate of permanent stroke associated with MICS utilizing femoral cannulation was twice as high as that observed in procedures utilizing conventional cannulation techniques (16). In contrast, other studies have demonstrated that transthoracic central aortic cannulation with antegrade perfusion offers superior reliability, safety, and a low complication rate (9, 15, 26). Furthermore, when the diameter of the FA is 7 mm or less or anatomical abnormalities are present, or in patients with atherosclerotic disease, it becomes crucial to explore alternative cannulation techniques, potentially even considering abandoning MICS approaches (15, 27). Also, FA cannulation can potentially result in a range of complications, including but not limited to arterial wall dissection, distal limb ischemia, and groin wound seromas (10, 11, 22). Subclavian artery cannulation emerges as a viable alternative, offering the benefit of maintaining blood flow without impeding the surgical field's visibility (15, 20). Nevertheless, this approach necessitates an additional incision, which adds complexity to the procedure. Furthermore, accessing the subclavian artery can pose challenges due to its deep-seated location and intricate anatomic surroundings (20, 28). The utilization of the EABO entails several risks, including the possibility of balloon rupture, lower extremity complications, and an increased stroke rate among individuals afflicted with aortoiliac disease (20). In addition, complete occlusion with an endo-aortic balloon cannot be guaranteed when the diameter of the aorta exceeds 40 mm (7). It has been reported that IJV-related complications range from 0.68% to 4.3%, including local hematomas, vascular injuries, thrombosis, and pulmonary complications (13). Additionally, while performing IJV cannulation for SVC drainage, factors such as an inadequate cannula size and retrograde flow can impede adequate drainage, elevating cerebral venous pressure and increasing the risk of cerebral edema, especially when there is a lack of vacuum assistance. Furthermore, another significant concern is the conversion rate from MICS to thoracotomy, reportedly ranging from 2.6% to 4%, primarily due to intraoperative challenging exposure, bleeding, and various other contributing factors (29).

Our approach to CPB and aortic occlusion for MICS involves transthoracic direct cannulation of the ascending aorta, SVC, and ETTC, utilizing an assist port positioned in the second intercostal space parasternal. Compared to FA cannulation and EABO, conventional cannulation and routine aortic occlusion techniques for antegrade perfusion are more physiological, significantly mitigating the likelihood of plaque embolization, iatrogenic aortic dissection, and relative potential complications (20, 22, 30). Central venous cannulation facilitates antegrade venous flow drainage, allows for the selection of larger cannula sizes, ensures adequate venous drainage, a clean and blood-free surgical field, and reduces the demands on the anesthesia team, costs, and IJV-related complications (7).

Our standard surgical approach for FVATCS includes a main incision of 40 mm, positioned at the 4th intercostal space, a 10 mm incision made in the same intercostal space to accommodate the camera port, and a 30 mm assist port for direct central cannulation and aortic occlusion. The main incision is situated either between the midclavicular and anterior axillary lines or between the anterior and midaxillary lines, depending on the surgical location of the heart. The assist port is placed in the second intercostal space, the parasternal. For aortic root surgery, an extended incision of 50–60 mm is made in the right second intercostal space, the parasternal. The camera port is situated laterally to the main incision within the second or third intercostal space. This FVATCS surgical platform can provide a direct, en-face view of the intracardiac structures and reduce interference within the extremely limited surgical field, especially in mitral valve repair, greatly enhancing visualization of the subvalvular apparatus, the secondary strut chords (6).

All the patients require potent analgesic medications in the initial 24–48 h post-surgery. Ropivacaine Hydrochloride Injection (20 ml) was diluted to 40 ml and administered intramuscularly around the incision site during the surgical procedure. Sufentanil infusion was administered for 24 h, then transitioned to oral gabapentin. If necessary, tramadol injections were administered. The postoperative pain scores ranged from 1 to 3.

FVATCS is an innovative surgical platform for MICS. Compared with direct vision in a minimal access approach, integrating video assistance visualization technology allows for smaller incisions and ensures better illumination, larger and clearer images, and effortless recording and broadcasting of the surgical procedure (14, 21). It provides surgeons with a high-resolution, real-time view of the surgical field, enabling them to perform more accurately and confidently, enhancing surgical precision and safety. Additionally, a crucial aspect of our FVATCS lies in minimizing the number of incisions made in the chest wall while ensuring safety and ease of operation. This reduction not only mitigates the direct trauma inflicted by the incision but also decreases the likelihood of bleeding complications resulting from the same incision.

In our surgical practice, we refrain from using FA cannulation, percutaneous cannulas, and endodontic balloon occlusion, reducing additional costs to the patient and dependency on the anesthesia team. This technique offers considerable advantages in developing countries. Also, this technique provides a potential solution for patients unsuitable for peripheral cannulation and enhances the inclusivity of MICS by accommodating patients with absolute contraindications to peripheral cannulation.

## Limitations

The limitations of our study reside primarily in its small sample size, uncontrolled, single-institutional, retrospective nature, and the absence of patient-matched controls comparing different cannulation techniques during the study period.

## Conclusions

The FVATCS platform integrates central cannulation antegrade perfusion with venous drainage, minimizing additional surgical procedures and cost, mitigating potential pitfalls, ensuring reasonable operation duration, enhancing the inclusivity of MICS, and minimizing complications, ultimately leading to a more favorable prognosis.

## Data Availability

The original contributions presented in the study are included in the article/Supplementary Material, further inquiries can be directed to the corresponding author.
