# Supplementary material for: Initial experience and favorable outcomes on cannulation strategies and surgical platform construction in fully video-assisted thoracoscopic cardiac surgery
Source: Front Cardiovasc Med. 2024 Aug 8;11:1414333. doi: 10.3389/fcvm.2024.1414333 (PMC11338890; doi:10.3389/fcvm.2024.1414333)
Supplement: Supplementary file 4 [file Datasheet1.docx]

Supplementary Material

# Supplementary Figures and Tables

## Supplementary Figures


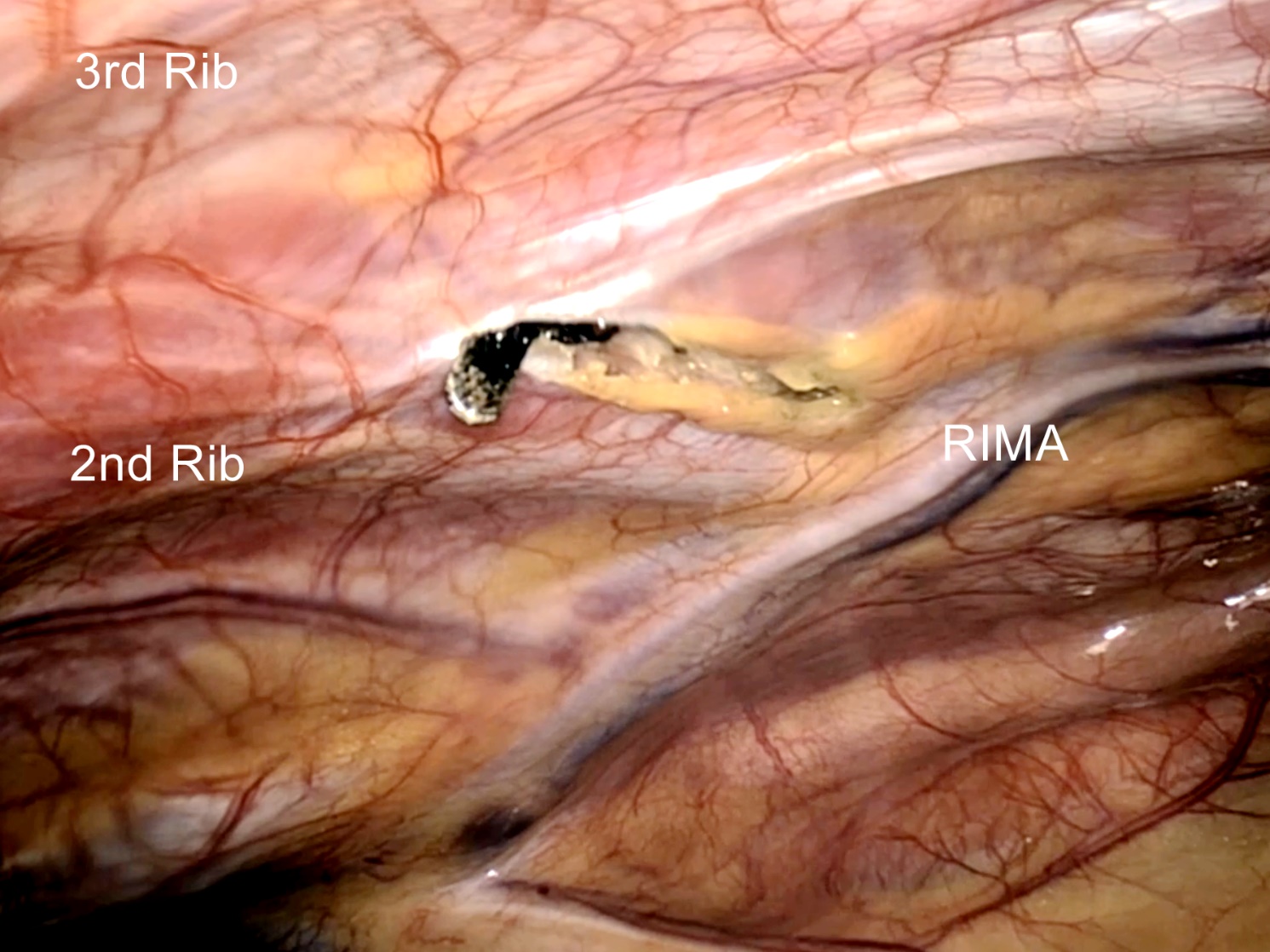


**Supplementary Figure 1.** Assist Port (Port 3) making under thoracoscopic guidance


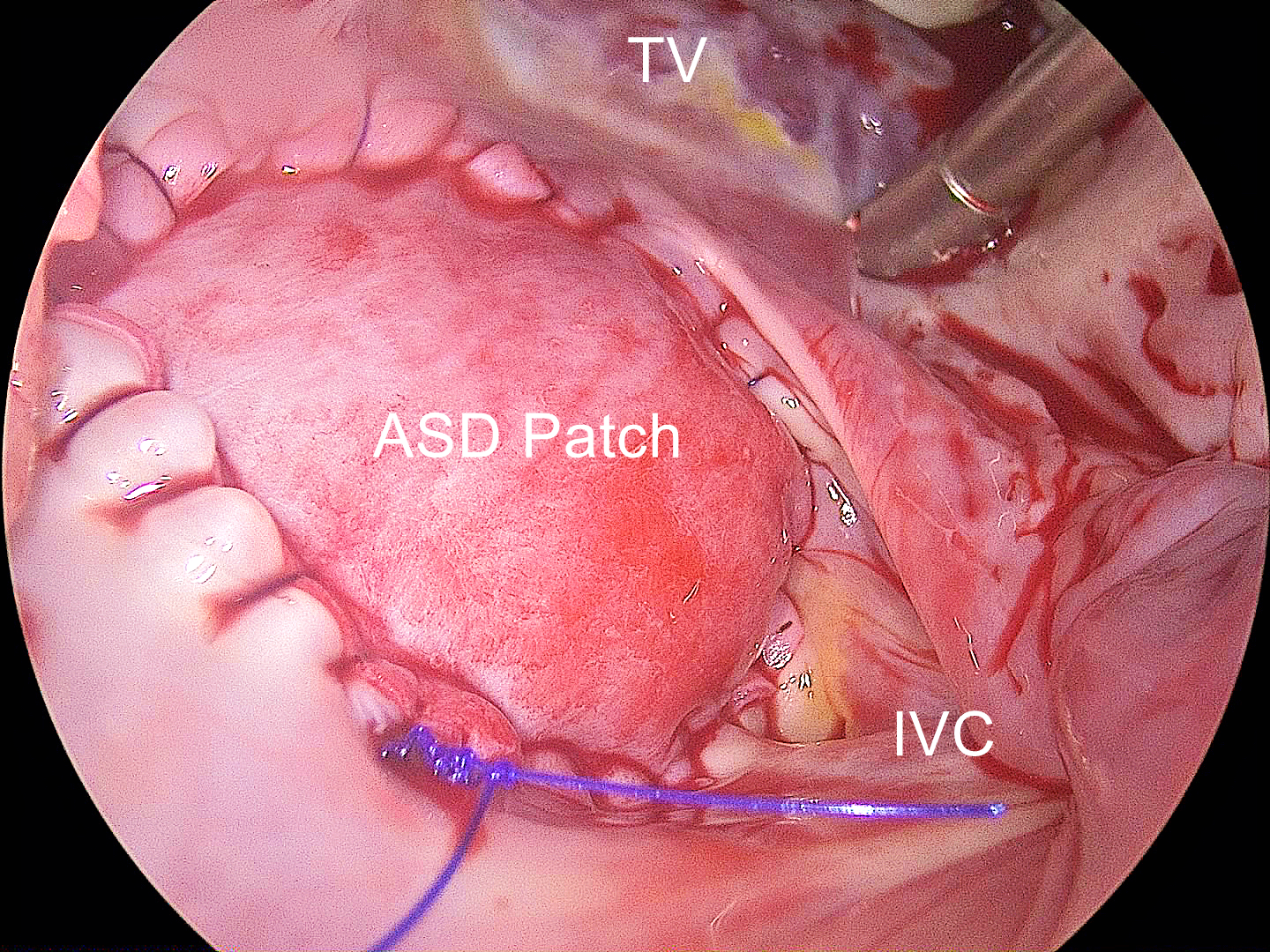


**Supplementary Figure 2.** Surgical View of ASD Repair


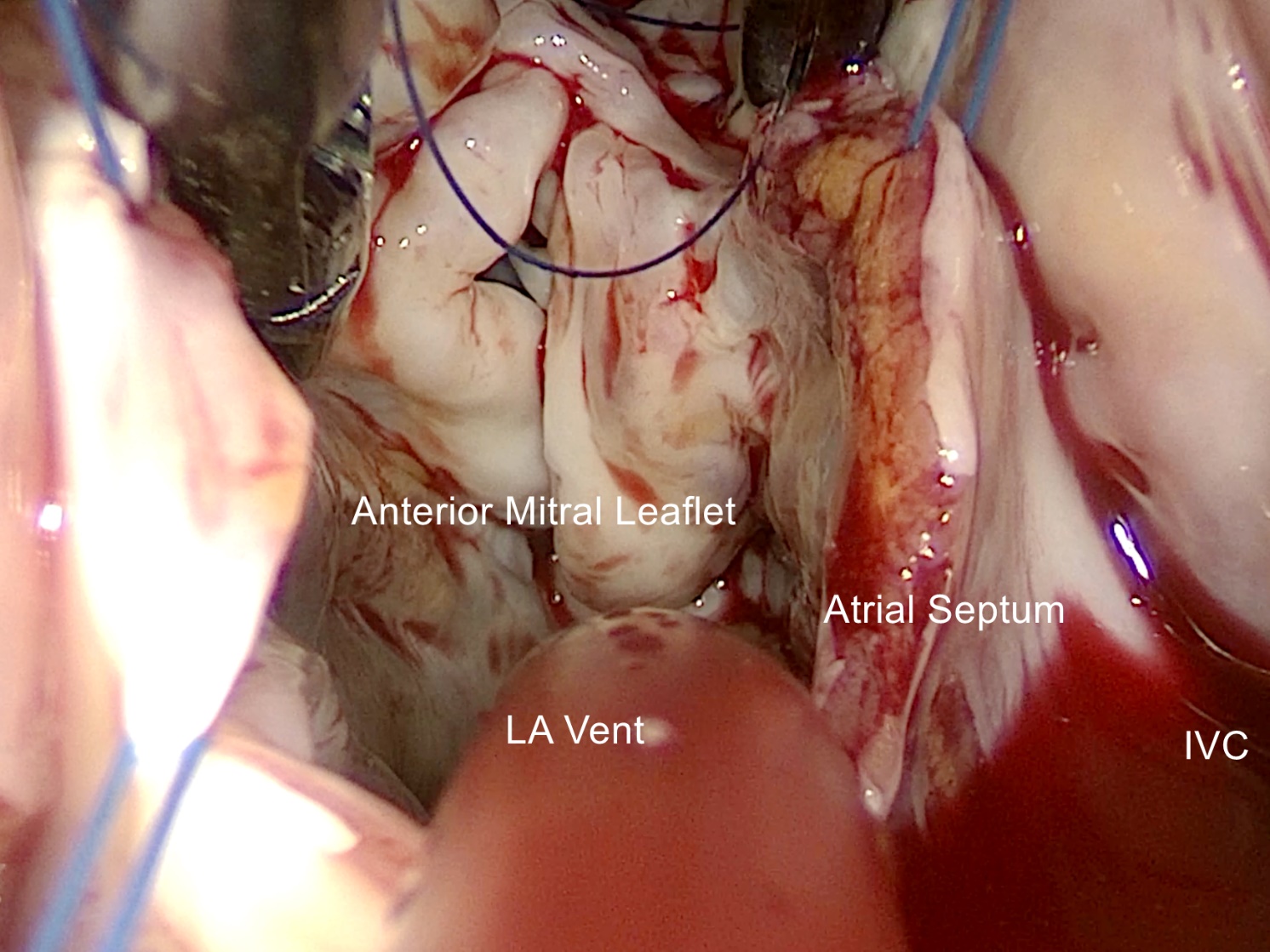


**Supplementary Figure 3.** Surgical View of PAVC Repair


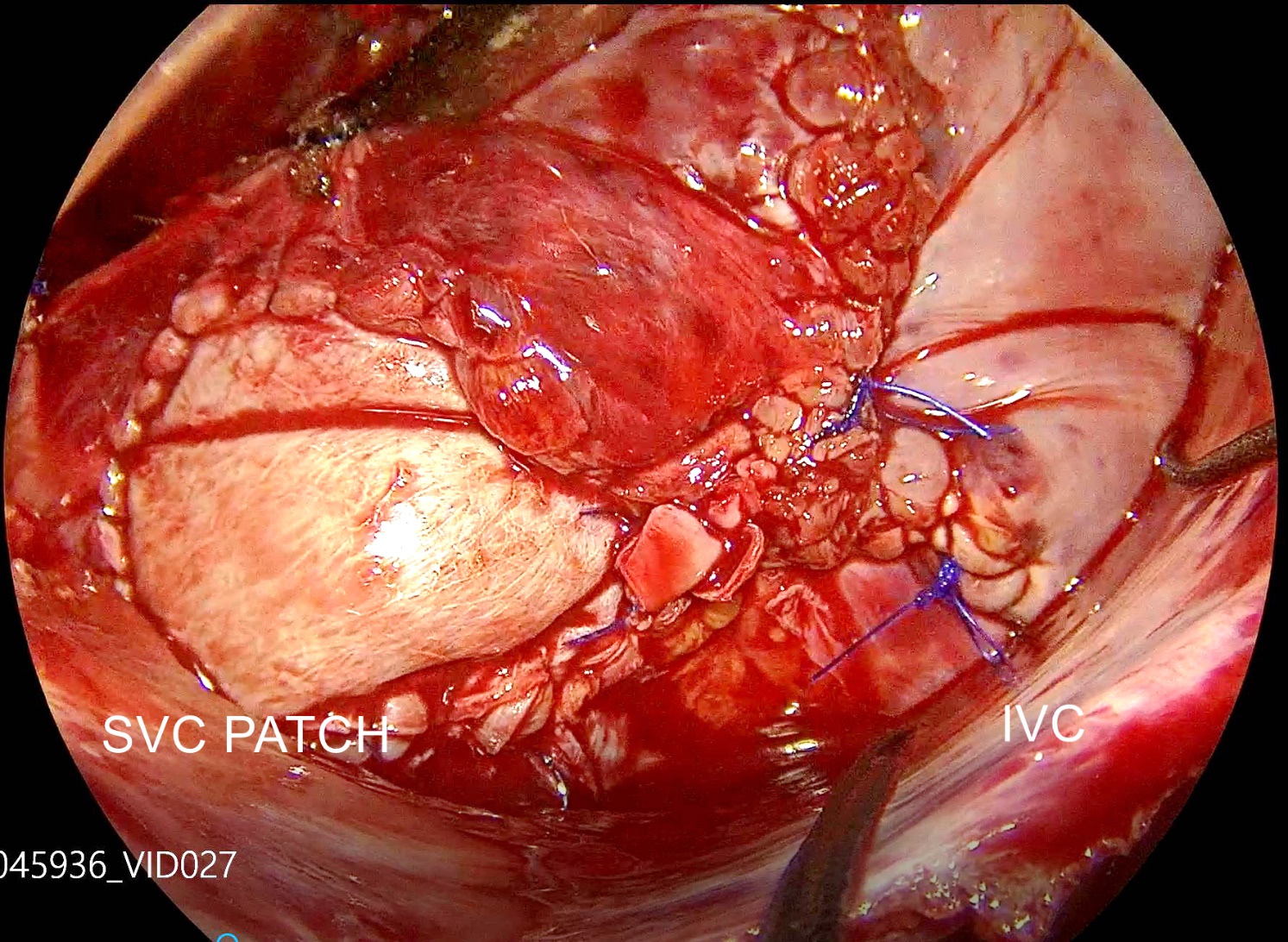


**Supplementary Figure 4.** Surgical View of PAPVC Repair


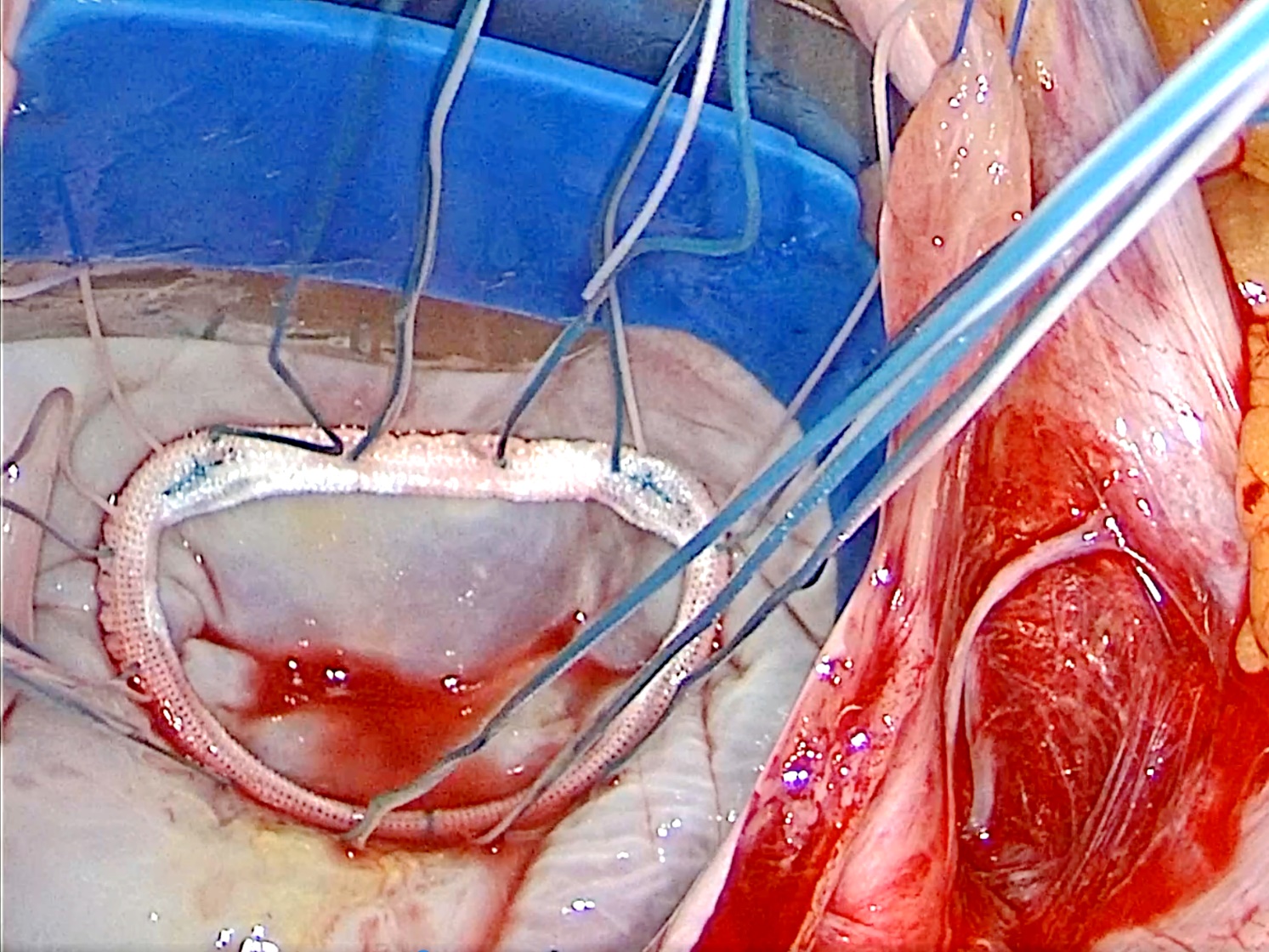


**Supplementary Figure 5.** Surgical View of Mitral Valve Repair


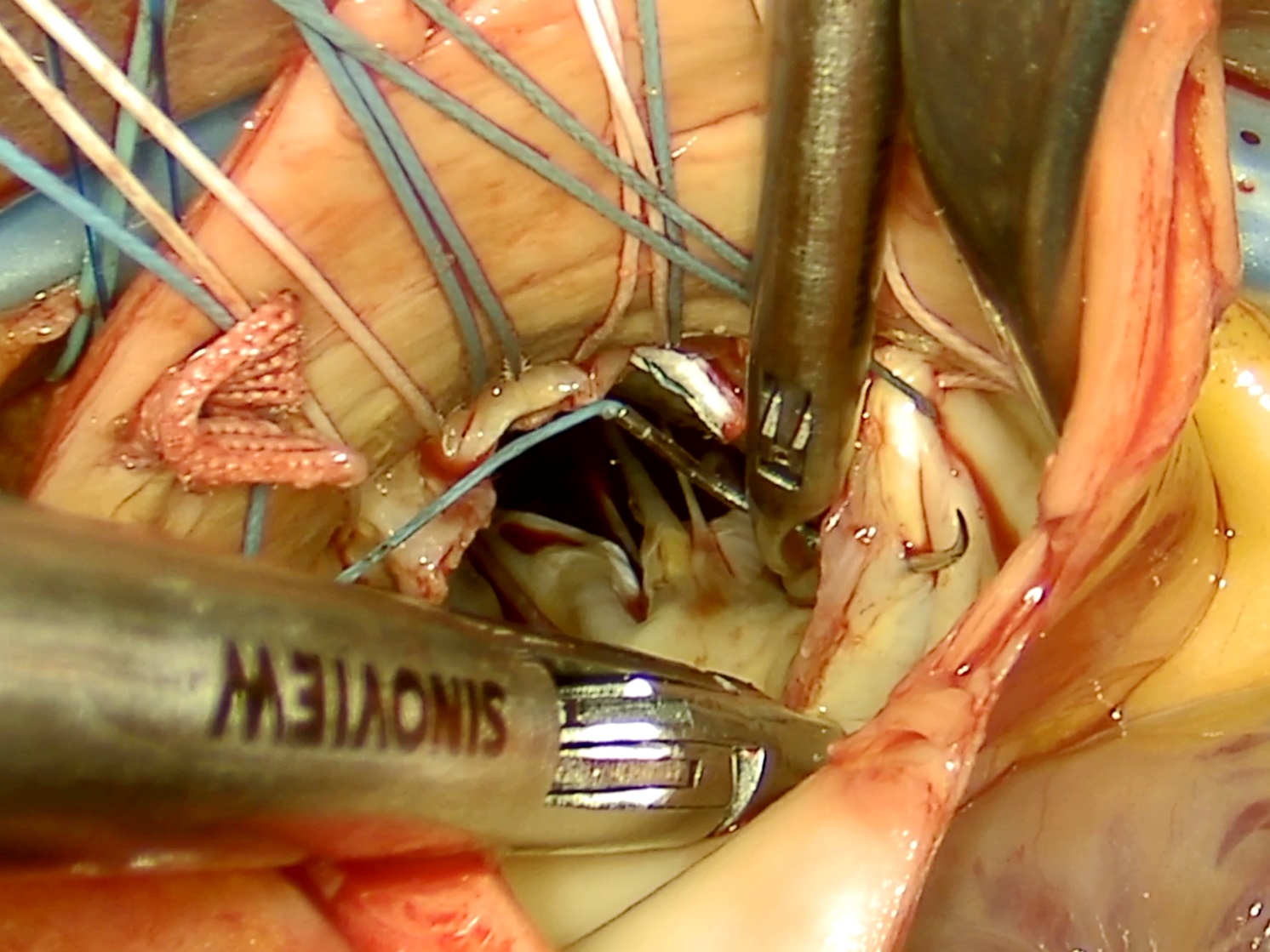


**Supplementary Figure 6.** Surgical View of Aortic Valve Replacement

**Supplementary Figure 1.** The figure legends are required to have the same font as the main text, 12 point normal Times New Roman, single spaced. Please use a single paragraph for each legend and prepare the figures keeping in mind the PDF layout.
